# Multi-strain probiotic reduces gastrointestinal side effects in women with elevated HOMA-IR index treated with metformin: a 12-week randomised controlled trial

**DOI:** 10.3389/fendo.2026.1765741

**Published:** 2026-03-03

**Authors:** Marzena Ratajczak, Agnieszka Bilska, Katarzyna Musialik, Karolina Skonieczna-Żydecka, Igor Łoniewski, Anna Gogojewicz, Joanna Karolkiewicz

**Affiliations:** 1 Department of Medical Biology, Poznan University of Physical Education, Poznan, Poland; 2 Department of Food and Nutrition, Poznan University of Physical Education, Poznan, Poland; 3 Department of Treatment of Obesity, Metabolic Disorders and Clinical Dietetics, Poznan University of Medical Sciences, Poznan, Poland; 4 Department of Biochemical Research, Pomeranian Medical University, Szczecin, Poland; 5 Sanprobi Sp. z o.o. Sp.K., Szczecin, Poland

**Keywords:** gastrointestinal side effects, HOMA-IR, metformin, probiotics, randomised controlled trial

## Abstract

**Background:**

Metformin is widely used as a first-line therapy for type 2 diabetes and increasingly prescribed off-label in women with elevated HOMA-IR indices, including those at risk of metabolic disorders. However, its clinical use is often limited by gastrointestinal (GI) adverse effects. The present study examined whether a multi-strain probiotic could enhance the metabolic effects of metformin and reduce GI side effects in women with newly identified elevated HOMA-IR.

**Methods:**

In this 12-week randomised, placebo-controlled, double-blind trial, 30 women aged 25–45 years with elevated HOMA-IR (≥2.5) and no diagnosis of diabetes were enrolled. All participants were prescribed metformin 1000 mg/day. They were randomised 1:1 to receive either a multi-strain probiotic (2 × 10^9^ CFU/day) or placebo. Outcomes included metabolic markers (glucose, insulin, HOMA-IR, RBP4, lipid profile, body composition) and self-reported GI symptoms.

**Results:**

After 12 weeks, the probiotic group reported significantly fewer GI symptoms compared with placebo, including lower frequency of abnormal stool consistency during abdominal pain (26% vs. 52%, p < 0.05), abnormal stool frequency (18% vs. 51%, p < 0.05), and hard or lumpy stools (Bristol types 1–2; 14% vs. 26%, p < 0.05). No significant between-group differences were observed for metabolic or anthropometric parameters. Both groups showed significant improvements over time in fasting glucose (time effect p < 0.05), HOMA-IR (p < 0.05), RBP4 (p < 0.05), and total cholesterol (p < 0.01), with no significant group × time interactions, indicating effects attributable to metformin rather than probiotic supplementation.

**Conclusion:**

Multi-strain probiotic supplementation did not enhance the metabolic efficacy of metformin in women with elevated HOMA-IR but significantly alleviated GI side effects. Probiotic co-administration may therefore improve tolerability and adherence to metformin therapy.

**Clinical trial registration:**

## 1 Introduction

Metformin exerts many of its glucose-lowering effects through the gastrointestinal tract, including modulation of bile acid metabolism, enteroendocrine signaling, and alterations in gut microbial composition, which may influence incretin secretion and intestinal glucose handling (1–7). However, despite growing mechanistic interest in gut–microbiota interactions, gastrointestinal intolerance remains one of the main clinical barriers to sustained metformin use, affecting approximately 20–30% of patients and frequently leading to dose reduction or treatment discontinuation (8). Therefore, strategies that improve gastrointestinal tolerability may have greater immediate clinical relevance than potential metabolic modulation, particularly in individuals treated in early stages of metabolic dysfunction.

Probiotic supplementation has been proposed as a potential approach to mitigate gastrointestinal side effects and, in some studies, to modestly improve glycaemic control. Meta-analyses and randomized trials report heterogeneous results regarding metabolic outcomes when probiotics are combined with metformin or used as adjunct therapy in insulin resistance and type 2 diabetes (9–12). In contrast, evidence regarding probiotic effects on metformin-associated gastrointestinal symptoms appears more consistent, with several clinical trials and meta-analyses demonstrating reductions in diarrhea, abdominal discomfort, and abnormal stool frequency (13, 14). These findings support the need to distinguish clinical tolerability outcomes from metabolic efficacy when evaluating adjunctive probiotic therapy during metformin treatment.

The primary outcome of the present trial was the change in gastrointestinal symptoms associated with metformin treatment, assessed using selected items from the Adult Rome IV Questionnaire.

Secondary outcomes included changes in metabolic and anthropometric parameters, specifically fasting glucose, fasting insulin, HOMA-IR, retinol-binding protein 4 (RBP4), lipid profile, body composition, and body mass index.

Accordingly, this randomized, double-blind, placebo-controlled trial aimed to determine whether multi-strain probiotic supplementation improves gastrointestinal tolerability of metformin and whether it exerts additional metabolic effects in women with elevated HOMA-IR.

## 2 Methods

### 2.1 Study design

This twelve-week, randomized, placebo-controlled, double-blind study compared the effects of a multi-strain probiotic versus a placebo in women with insulin resistance who were recommended by an internal medicine physician to take 1000 mg of metformin (500 mg twice daily, during or after meals). The study was registered on ClinicalTrials.gov (NCT06092060) on October 23, 2023. Blood samples were collected for biochemical analyses before and after the 12-week intervention, body composition assessments were performed, and participants completed questionnaires regarding gastrointestinal symptoms.

### 2.2 Participants

Participants were recruited through advertisements on the Polish Insulin Resistance Foundation–Healthy Diet and Healthy Life website. Eligible participants were included in this study if they met the following criteria: female, aged 25–45 years, BMI 25-39.9 kg/m², and insulin resistance (based on medical qualification: HOMA-IR and insulin curve were considered). All patients were required to be treated with metformin (1000 mg/daily). Patients were excluded if they had received metformin treatment before the study, had type 1 or type 2 diabetes, PCOS or had any acute or chronic clinically apparent inflammatory processes (connective tissue and joint diseases, respiratory tract inflammatory processes, urogenital inflammatory processes, inflammatory processes in the head and neck area), acute infection within the last month, cancer, alcohol abuse, drug addiction, immunodeficiency, chronic treatment with immunosuppressants, glucocorticoids, or use of antibiotics and non-steroidal anti-inflammatory drugs, steroids, antifungals within the last 30 days; daily consumption of synbiotics, prebiotics, and/or probiotics within the past 90 days; or travel to tropical countries in the last 4 weeks before the intervention. Other conditions that could pose any risk to the patient during the observation period were also exclusionary.

Consecutive potentially eligible patients attending the Department of Treatment of Obesity, Metabolic Disorders and Clinical Dietetics, Poznan University of Medical Sciences were recruited by a diabetologist for participation in this study.

All volunteers were introduced to the study’s purpose and protocols and provided written consent to participate. During the screening visit, the inclusion/exclusion criteria were confirmed based on interviews and medical records, and baseline demographic data were collected.

**Figure 1:**
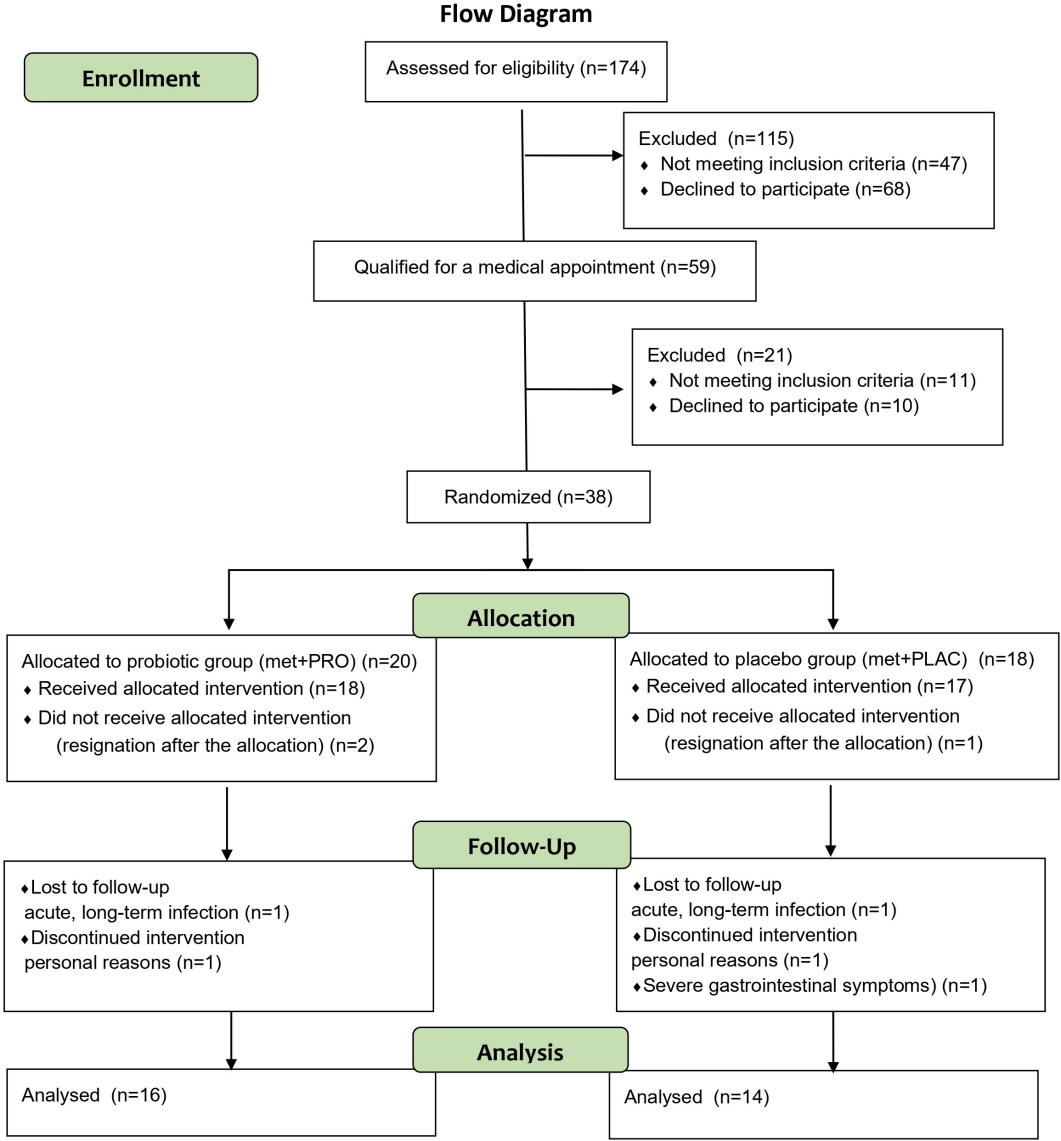
The study flow diagram.

The study flow diagram is presented in **Figure 1**.

Before participation, all candidates underwent medical qualification. Patients from Poznan University of Medical Sciences, who met the inclusion criteria and were prescribed metformin by an internal medicine specialist, were invited to participate in the scientific project. A complete medical history and physical examination were conducted at the Department of Treatment of Obesity, Metabolic Disorders, and Clinical Dietetics of the Poznan University of Medical Sciences. Volunteers were informed about the purpose and methodology of the study and provided written informed consent.

Before starting the study, fasting venous blood samples were collected from all participants to measure glucose and insulin levels and calculate the HOMA-IR index (Matthews et al., 1985). Patients whose fasting glucose levels were between 3.0 and 25.0 mmol/L and fasting insulin levels between 3 and 55 mU/mL had their glycated hemoglobin (HbA1c) measured to exclude diabetes or prediabetes, based on the following criteria (Carson et al., 2010):

- Type 2 diabetes was diagnosed if one or more of the following conditions were met: HbA1c ≥ 6.5%, fasting glucose ≥ 7 mmol/L (126 mg/dL), self-reported diabetes, or current use of diabetes medication (oral hypoglycemics and/or insulin).
- Prediabetes was confirmed if one or more of the following conditions were met: HbA1c ≥ 6% but < 6.5%, or fasting glucose ≥ 5.5 mmol/L (100 mg/dL) but < 7.0 mmol/L (126 mg/dL), with no self-reported diabetes and no diabetes medication use.

Thirty women diagnosed with insulin resistance, aged 25 to 45, were randomized into two groups using randomization software (www.randomizer.org), by the probiotic manufacturer in a 1:1 (probiotic: placebo) ratio. Allocation concealment was ensured with sequentially numbered drug containers of identical appearance. Blinding included both the authors of the study and the participants. The distribution of participants between the probiotic group (n = 16) and the placebo group (n = 14) in the final study group was impacted by elevated dropout rates and the presence of outliers, predominantly in the treatment group.

### 2.3 Interventions

The probiotic group (Met+PROB) received two capsules twice daily (in the mornings and before bedtime) containing a lyophilized powder of the following probiotic strains: *Bifidobacterium lactis W52, Levilactobacillus brevis W63, Lacticaseibacillus casei W56, Lactococcus lactis W19, Lactococcus lactis W58, Lactobacillus acidophilus W37, Bifidobacterium bifidum W23, Bifidobacterium lactis W51, and Ligilactobacillus salivarius W24* (Sanprobi Sp. z o.o. Sp. k., Szczecin, Poland) at a daily dose of 2 × 10^9^ colony-forming units (CFU), starting the day they began taking metformin (1000 mg/day) for 12 weeks. The probiotic preparation was microbiologically controlled to ensure consistency in strains and CFU counts throughout the study. Supplemented probiotics were considered safe and well-tolerated in the healthy population, and the tested product held the necessary safety certificates.

The placebo group (Met+PLAC) received two capsules twice daily containing only the probiotic carrier, corn starch and maltodextrins. All participants were instructed to take two capsules twice daily, once before breakfast and once before bedtime, starting on the same day they began metformin (1000 mg/day). Participants returned after four weeks to submit empty and partially used blister packs for compliance monitoring. They were also instructed not to change their usual physical activity or diet. Any side effects from metformin and/or the probiotic were to be recorded or reported by phone.

After initial qualification (based on inclusion and exclusion criteria), each participant underwent two sets of tests: at baseline (PRE) and after 12 weeks (POST).

### 2.4 Measurements

Measurements were performed at the University of Physical Education in Poznan and Poznan University of Medical Sciences.

#### 2.4.1 Dietary pattern and gastrointestinal side effects

A medical interview was conducted with each patient. Prior to the initiation of the clinical trial, an informational meeting was held, and participants were instructed to maintain their usual dietary patterns and physical activity levels during the study and to report any dietary and physical activity changes, the emergence of exclusion criteria, or potential adverse events. To ensure comparable dietary habits, a dietitian assessed the daily meal distribution, total caloric intake, and qualitative composition based on a detailed dietary interview and food diary analysis. A standardized diet plan was established for all participants. Dietary intake of energy was verified using 7-day food records at the beginning and end of the 12-week study period, with the Nuvero software to ensure diet consistency. An evaluation of gastrointestinal disorders was conducted using the Adult Rome IV Questionnaire, which included selected questions addressing the most common gastrointestinal disorders, such as functional dyspepsia, as well as characteristics of abdominal pain, the relationship of symptoms to bowel movements, patterns of bowel movements, and other associated symptoms.

#### 2.4.2 Anthropometric and body composition indicators

Anthropometric measurements were performed in the morning, in light clothing and without shoes. Body weight and height were measured using a medical scale with a stadiometer (seca 285, Hamburg, Germany), with accuracies of 0.1 kg and 0.5 cm, respectively. BMI was calculated from body weight and height using a standard formula. Body composition was assessed using dual-energy X-ray absorptiometry (DXA; Lunar Prodigy device, GE Healthcare, Chicago, USA). Patients received full instructions on the body composition analysis procedure and were instructed not to engage in vigorous physical activity for 24 hours prior to the measurement. Total tissue fat (%), visceral adipose tissue (kg), and total lean mass (kg) were determined using a standard scanning mode, with an absorbed radiation dose of 0.4 µGy.

#### 2.4.3 Biochemical markers

Venous blood samples were collected in the morning after 12 hours of fasting, both before and after the 3-month intervention. The blood samples were centrifuged and stored at −80 °C until analysis. Serum samples were analyzed for glucose, HbA1c, thyrotropin (TSH), and lipid profile, including total cholesterol, high-density lipoprotein (HDL), and triglyceride levels, using a Dimension EXL with LM Integrated Chemistry System Analyzer (Siemens, Newark, NJ, USA). The concentration of low-density lipoprotein (LDL) cholesterol was calculated using the Friedewald equation. RBP4 was measured using a test produced by Immun Diagnostik (Germany). Serum insulin was measured using an immunoradiometric assay (DIAsource Immunoassays S.A., Nivelles, Belgium), with a manufacturer-reported minimum detectable value of 1.0 µIU/mL. IR was estimated using the following formula: HOMA-IR index = fasting insulin [mU/L] × fasting glucose [mmol/L]/22.5.

### 2.5 Statistical analysis

The sample size was calculated for HOMA-IR and was determined based on a similar study (15) using GPower version 3.1.9.4. Sample size analysis indicated that a minimum of 12 subjects per group was required to achieve at least 80% power to detect a statistically significant intervention effect at α = 0.05. To confirm the similarity between the Met+PROB and Met+PLAC groups, tests for comparisons of independent groups were performed before the intervention, using either the Student’s t-test or the Mann–Whitney U test, depending on the distribution of the variable. Anthropometric variables, dietary intake, and biochemical indicators were compared using two-way repeated measures ANOVA. For statistically significant results, Bonferroni *post hoc* tests were additionally conducted. To compare responses regarding gastrointestinal side effects, mean percentage values between groups were compared separately before and after the intervention. Quantitative variables were analyzed using the Student’s t-test or the Mann–Whitney U test (depending on variable distribution), and qualitative variables using Pearson’s Chi-square (χ²) test. The eta squared coefficient (η^2^) is presented as an indicator of effect size.

## 3 Results

The study flow diagram is presented in **Figure 1**. The pre-study demographic profiles and characteristics of the patients in both groups exhibited no significant differences. All patients were female. Based on the provided baseline characteristics, the probiotic group (Met+PROB) and the placebo group (Met+PLAC) appear to be well matched across several parameters (**Table 1**). Compliance with the requirement to take metformin and probiotic or placebo capsules was 100%.

**Table 1:** Pre-study demographic and clinical characteristics of patients.

Variable	Met+PROB N=16	Met+PLAC N=14	P-value
Age [years]	36.7 ± 5.7	33.1 ± 4.6	0.06329^b^
Body mass [kg]	83.2 ± 10.4	86.6 ± 10.6	0.39271^a^
BMI [kg/m^2^]	30.0 ± 3.5	31.0 ± 3.2	0.42146^a^
Total Fat Mass [kg]	35.1 ± 7.8	37.2 ± 6.3	0.37517^a^
Glucose [mmol/L]	5.3 ± 0.3	5.4 ± 0.4	0.66827^a^
Insulin [µU/ml]	13.3 ± 4.7	15.0 ± 6.7	0.80299^b^
HOMA-IR	3.2 ± 1.1	3.6 ± 1.5	0.66248^b^
Thyrotropin [uIU/mL]	1.9 ± 0.7	2.2 ± 1.5	0.45306^a^

^a^Student t-test, ^b^U Mann-Whitney test.

The effects of the 12-week probiotic supplementation during metformin therapy on anthropometric measurements and body composition are presented in **Table 2**. Repeated measures ANOVA did not reveal any group × time interactions, and the analysis of anthropometric and body composition data showed no significant differences between Met+PROB and Met+PLAC before or after the intervention.

**Table 2:** Anthropometric and body composition before and after 12 weeks.

Variable		Met+PROB n=16		Met+PLAC n=14			ANOVA		
		PRE	POST	PRE	POST		Group effect	Time effect	Interaction
BMI [kg/m^2^]	Mean SD	30.0 3.5	30.2 3.5	31.0 3.2		31.5 3.6	F=0.32 ƞ^2^ = 0.01	F=2.10 ƞ^2^ = 0.07	F=0.98 ƞ^2^ = 0.04
Total Fat Mass [kg]	Mean SD	35.1 7.8	36.1 6.8	37.2 6.3		40.3 8.0	F=0.96 ƞ^2^ = 0.04	F=0.97 ƞ^2^ = 0.04	F=1.16 ƞ^2^ = 0.04
Visceral Adipose Tissue [kg]	Mean SD	1.2 0.5	1.2 0.5	1.1 0.4		1.1 0.5	F=0.12 ƞ^2^ = 0.00	F=0.92 ƞ^2^ = 0.03	F=0.00 ƞ^2^ = 0.00
Total Lean Mass [kg]	Mean SD	44.7 3.9	44.8 4.7	44.7 4.2		45.0 4.1	F=0.01 ƞ^2^ = 0.00	F=0.70 ƞ^2^ = 0.03	F=0.10 ƞ^2^ = 0.00

Values are mean ± SD; PRE = baseline; POST = after 12 weeks.

The characteristics of the diet are presented in **Table 3**. Diet is a significant factor that modifies the gut microbiome; therefore, an analysis of dietary habits was conducted. The comparative analysis of the patients’ diets revealed no significant differences between the groups in terms of energy and main nutrients. Both groups had overlapping ranges in their intake. The estimated energy and fiber intakes showed some differences in mean post-study values (1,780 ± 467.5 kcal/day in the probiotic group vs. 1,653.3 ± 581.4 kcal/day in the placebo group, and 27.5 ± 6.8 vs. 21.9 ± 7.6 g/day, respectively); however, the ranges overlapped, suggesting individual variability but no clear distinction between the groups.

**Table 3:** Dietary intake before and after 12 weeks.

Variable		Met+PROB n=16		Met+PLAC n=14		ANOVA		
		PRE	POST	PRE	POST	Group effect	Time effect	Interaction
Energy intake [kcal/day]	Mean	1711.4	1780.0	1705.5	1653.3	F=0.18. ƞ^2^ = 0.01	F=0.01 ƞ^2^ = 0.00	F=0.53 ƞ^2^ = 0.02
	SD	457.9	467.5	324.7	581.4			
Protein intake [g/day]	Mean	82.7	84.5	78.0	77.7	F=0.71 ƞ^2^ = 0.03	F=0.02 ƞ^2^ = 0.00	F=0.07 ƞ^2^ = 0.00
	SD	19.1	24.7	13.5	27.3			
Fat intake [g/day]	Mean	64.8	67.8	69.6	64.6	F=0.00 ƞ^2^ = 0.00	F=0.04 ƞ^2^ = 0.00	F=0.96 ƞ^2^ = 0.03
	SD	22.9	21.5	17.1	24.3			
Carbohydrates intake [g/day]	Mean SD	208.4	219.9	200.4	197.2	F=0.55 ƞ^2^ = 0.02	F=0.06 ƞ^2^ = 0.00	F=0.67 ƞ^2^ = 0.02
		61.6	55.6	45.1	66.4			
Sugar intake [g/day]	Mean SD	57.3	61.8	53.5	54.7	F=0.41 ƞ^2^ = 0.02	F=0.47 ƞ^2^ = 0.02	F=0.19 ƞ^2^ = 0.01
		24.3	26.0	23.3	24.5			
Dietary fiber [g/day]	Mean SD	25.7	27.5	23.3	21.9	F=3.34 ƞ^2^ = 0.11	F=0.06 ƞ^2^ = 0.00	F=2.17 ƞ^2^ = 0.08
		7.0	6.8	4.2	7.6			

Values are mean ± SD; PRE = baseline; POST = after 12 weeks.

The effects of 12 weeks of supplementation with probiotics or placebo during metformin therapy on biochemical indicators are presented in **Table 4**. Repeated ANOVA analysis did not reveal any interactions between group and time for the measured indicators. Over the 12-week period, significant time effects were observed for glucose, HOMA-IR, and RBP4 (p < 0.05), as well as for total cholesterol levels (p < 0.01). Compared with pre-study values, both groups showed post-study decreases in glucose (by 0.1 mmol/L in the probiotic group and 0.5 mmol/L in the placebo group), RBP4 (by 1.4 and 7.9 μg/mL, respectively), total cholesterol (by 0.6 and 0.5 mmol/L, respectively), and HOMA-IR (by 0.7 and 0.8 points, respectively). The results obtained after the therapy indicate a significant improvement in glucose and lipid metabolism indicators in both examined groups, which was independent of the probiotic supplementation, suggesting a positive effect of metformin on the health of the women studied.

**Table 4:** Biochemical indicators before and after 12 weeks.

Variable		Met+PROB n=16		Met+PLAC n=14		ANOVA		
		PRE	POST	PRE	POST	Group effect	Time effect	Interaction
Glucose [mmol/L]	Mean SD	5.3 0.3	5.2 0.7	5.4 0.4	4.9 0.8	F=0.54 ƞ^2^ = 0.02	F=6.45 ƞ^2^ = 0.19*	F=1.80 ƞ^2^ = 0.06
Insulin [µU/mL]	Mean SD	13.3 4.7	10.5 3.9	15.0 6.7	12.6 5.2	F=1.91 ƞ^2^ = 0.06	F=4.01 ƞ^2^ = 0.13	F=0.01 ƞ^2^ = 0.00
HOMA-IR	Mean SD	3.2 1.1	2.5 1.1	3.6 1.5	2.8 1.4	F=1.46 ƞ^2^ = 0.05	F=4.54 ƞ^2^ = 0.14*	F=0.02 ƞ^2^ = 0.00
RBP4 [μg/mL]	Mean SD	23.3 11.7	21.9 11.4	30.1 8.5	22.2 14.2	F=0.85 ƞ^2^ = 0.03	F=5.30 ƞ^2^ = 0.16*	F=2.61 ƞ^2^ = 0.09
Total cholesterol [mmol/L]	Mean SD	5.4 1.0	4.8 1.0	4.8 0.8	4.3 0.8	F=4.39 ƞ^2^ = 0.14*	F=7.67 ƞ^2^ = 0.22**	F=0.16 ƞ^2^ = 0.01
HDL-cholesterol [mmol/L]	Mean SD	1.5 0.4	1.3 0.2	1.3 0.2	1.3 0.2	F=2.48 ƞ^2^ = 0.08	F=2.13 ƞ^2^ = 0.07	F=1.83 ƞ^2^ = 0.06
LDL-cholesterol [mmol/L]	Mean SD	3.8 0.8	3.5 0.8	3.0 0.8	2.9 0.8	F=6.28 ƞ^2^ = 0.19*	F=2.35 ƞ^2^ = 0.08	F=0.50 ƞ^2^ = 0.02
Triglycerides [mmol/L]	Mean SD	1.4 0.5	1.5 0.3	1.8 0.8	1.7 0.5	F=2.92 ƞ^2^ = 0.10	F=0.13 ƞ^2^ = 0.00	F=0.47 ƞ^2^ = 0.02

Values are mean ± SD; PRE = baseline; POST = after 12 weeks; *p < 0.05; **p < 0.01.

The effects of 12-week supplementation with probiotics or placebo during metformin therapy on gastrointestinal side effects are presented in **Table 5**. Among the women with insulin resistance surveyed, no significant differences were observed between the groups in the frequency of responses to questions regarding the occurrence of functional digestive disorders before the intervention, with the exception of the question: ‘How often were stools mushy or watery (Type 6 or 7)?’, (13% ± 19% in Met+PROB and 27% ± 23% in Met+PLAC, p = 0.029). However, after 12 weeks of probiotic treatment, the study group Met+PROB reported fewer gastrointestinal adverse effects associated with metformin treatment compared to the placebo group. After the intervention, among patients taking probiotics, the occurrence of softer or harder stools than usual when abdominal pain was present was significantly lower compared to the placebo group: 26% ± 26% in Met+PROB vs. 52% ± 21% in Met+PLAC (p = 0.030). Similarly, abnormal stool frequency was less common: 18% ± 19% in Met+PROB vs. 51% ± 28% in Met+PLAC (p = 0.010), and the proportion of hard or lumpy stools was also lower: 14% ± 24% in Met+PROB vs. 26% ± 20% in Met+PLAC (p = 0.017).

**Table 5:** Gastrointestinal side effects before and after 12 weeks.

Symptom (Rome IV)	PRE		POST	
	Met+PROB	Met+PLAC	Met+PROB	Met+PLAC
Softer/harder stools when pain present (%)	50% 0.37	63% 0.25	26% 0.26	52% 0.21
	P = 0.30312^b^		P = 0.02978^a^	
Abnormal stool frequency with pain (%)	36% 0.39	48% 0.29	18% 0.19	51% 0.28
	P = 0.26567^b^		P = 0.00994^a^	
Hard or lumpy stools (Type 1–2) (%)	19% 0.13	17% 0.18	14% 0.24	26% 0.20
	P = 0.43966^b^		P = 0.01703^b^	
Mushy or watery stools (Type 6–7) (%)	13% 0.19	27% 0.23	12% 0.17	20% 0.14
	P = 0.02872^b^		P = 0.06475^b^	

All questions refered to the past 3 months.

Only significant or near-significant results presented for clarity.

^a^ Student t-test, ^b^ U Mann-Whitney test, ^c^ Pearson Chi^2^.

## 4 Discussion

This randomized, double-blind, placebo-controlled trial demonstrated that multi-strain probiotic supplementation significantly reduced gastrointestinal adverse effects associated with metformin initiation in women with elevated HOMA-IR, while no additional metabolic benefits beyond those attributable to metformin were observed. Improvements in glycaemic and lipid parameters occurred in both groups and were consistent with the known pharmacological effects of metformin, whereas probiotic co-administration primarily influenced gastrointestinal tolerability.

### 4.1 Gastrointestinal tolerability and probiotic effects

The primary outcome of this study was improvement in gastrointestinal symptoms assessed by the Rome IV questionnaire. After 12 weeks, participants receiving probiotics reported significantly lower frequencies of abnormal stool consistency and abnormal stool frequency during episodes of abdominal pain compared with the placebo group. These findings are clinically relevant, as gastrointestinal intolerance remains one of the most common reasons for metformin discontinuation and dose reduction, negatively affecting adherence and long-term metabolic outcomes (8).

Our results are consistent with recent clinical trials and meta-analyses indicating that probiotic supplementation can reduce metformin-associated gastrointestinal adverse events. A 2024 meta-analysis by Szymczak-Pajor et al. including randomized trials in patients with type 2 diabetes demonstrated significant reductions in diarrhea and abdominal discomfort with probiotic co-administration (14). Similarly, the ProGasMet trial reported improved gastrointestinal tolerability in metformin-intolerant patients receiving probiotic therapy (13). These data support the hypothesis that probiotics may enhance intestinal barrier function, normalize intestinal motility, and reduce luminal inflammation, thereby mitigating drug-related gastrointestinal disturbances.

### 4.2 Strain-specific considerations

The probiotic preparation used in the present study contained nine strains belonging to the genera *Lactobacillus*, *Bifidobacterium*, and *Lactococcus*. Several of these strains have previously been associated with modulation of intestinal permeability, short-chain fatty acid production, and regulation of bowel motility, which may explain the observed improvement in stool-related symptoms (16).

Strains of *Lactobacillus acidophilus*, *Lactobacillus brevis*, and *Lactobacillus casei* have been shown to support mucosal barrier integrity and reduce visceral hypersensitivity in functional bowel disorders, potentially contributing to reduced abdominal discomfort and improved stool consistency (17, 18). *Bifidobacterium lactis* and *Bifidobacterium bifidum* are frequently associated with enhanced production of acetate and lactate, which can stabilize intestinal transit and promote epithelial health (19).

*Lactococcus lactis* strains, while traditionally associated with dairy fermentation, have also demonstrated immunomodulatory properties that may attenuate low-grade intestinal inflammation (20). Importantly, the total probiotic dose used in this study (2 × 10^9^ CFU/day) was moderate compared with doses used in some metabolic studies. While this dose appears sufficient to influence gastrointestinal symptoms, it may be insufficient to induce measurable systemic metabolic effects, particularly in the context of concurrent metformin therapy, which itself strongly alters gut microbiota composition and host metabolism (9, 11, 12).

### 4.3 Lack of additional metabolic benefit

The results of studies investigating the effects of probiotic supplementation on glucose and lipid metabolism in patients with insulin resistance remain inconclusive. While some studies report beneficial effects, others—including the present study—have not demonstrated significant changes. For instance, a meta-analysis of 12 randomized controlled trials found that probiotic supplementation was associated with a significant reduction in glycated hemoglobin (HbA1c) levels and fasting insulin concentrations in patients with type 2 diabetes. However, the effects on fasting glucose levels, C-reactive protein (CRP), and lipid profiles were either non-significant or exhibited considerable heterogeneity (21). Despite theoretical synergy between probiotics and metformin on gut-mediated metabolic pathways, in our study no significant probiotic-related improvements in fasting glucose, insulin, HOMA-IR, RBP4, lipid profile, or body composition were observed. These findings align with several recent randomized trials reporting neutral metabolic effects of probiotic supplementation when combined with standard glucose-lowering therapy (21, 22).

Recent meta-analyses also indicate heterogeneity in metabolic responses to probiotics. A 2023 systematic review, which included 14 randomized controlled trials involving 1009 patients by Memon et al. found modest reductions in fasting glucose and HbA1c when probiotics were combined with metformin. Importantly, the frequency of gastrointestinal adverse effects was also found to be lower in the group receiving probiotics compared to the group receiving metformin alone (9). A 2025 network meta-analysis comparing different probiotic formulations in type 2 diabetes suggested that metabolic benefits are strain- and dose-dependent and may require higher CFU doses and longer treatment durations (12). Moreover, baseline metabolic severity appears to influence responsiveness, with larger effects observed in patients with established diabetes compared with those with early insulin resistance.

In the present study, participants had elevated HOMA-IR but normal glucose and HbA1c levels, which may have limited the potential for detecting metabolic improvement. Additionally, both groups demonstrated significant time effects for glucose, HOMA-IR, RBP4, and total cholesterol, reflecting the expected therapeutic response to metformin initiation. Any incremental probiotic effect may therefore have been masked by the dominant pharmacological action of metformin.

### 4.4 Clinical implications

From a clinical perspective, improving tolerability of metformin may be as important as enhancing its metabolic efficacy, particularly during treatment initiation. Gastrointestinal side effects are a major contributor to poor adherence, and even mild symptoms may lead to dose reduction or discontinuation. The observed reduction in stool-related symptoms suggests that probiotic co-administration could represent a simple, low-risk strategy to improve patient comfort and persistence with therapy, especially in individuals initiating metformin for insulin resistance or early metabolic dysfunction.

### 4.5 Strengths and limitations

Strengths of this study include the randomized, double-blind, placebo-controlled design, standardized assessment of gastrointestinal symptoms using Rome IV criteria, objective measurement of metabolic outcomes, and good adherence to both metformin and study supplementation.

Several limitations should be acknowledged. The sample size was small, limiting statistical power to detect small-to-moderate metabolic effects. Gastrointestinal outcomes were based on self-reported questionnaires, which may be subject to reporting bias. No gut microbiota, short-chain fatty acid, or bile acid analyses were performed, precluding mechanistic interpretation of probiotic effects. Additionally, metformin and probiotics were initiated simultaneously, making it difficult to distinguish whether probiotics prevented symptom development or merely attenuated ongoing intolerance. Finally, the study population was limited to women of reproductive age, which may restrict generalizability to men and older adults.

### 4.6 Future directions

Future trials should include larger sample sizes, longer follow-up periods, and incorporation of microbiome and metabolite analyses to clarify mechanistic pathways. Stratification by baseline gastrointestinal sensitivity and insulin resistance severity may help identify subgroups most likely to benefit from probiotic co-administration. Comparative studies of different probiotic formulations and higher CFU doses may further optimize adjunctive strategies to improve metformin tolerability and metabolic outcomes.

## 5 Conclusion

In women with elevated HOMA-IR treated with metformin, multi-strain probiotics significantly reduced gastrointestinal side effects but did not enhance metabolic efficacy. Probiotic supplementation may therefore be a valuable adjunct to improve adherence to metformin therapy. Larger and longer studies are warranted, particularly in populations with PCOS or prediabetes, to clarify potential metabolic benefits.

## Data Availability

The raw data supporting the conclusions of this article will be made available by the authors, without undue reservation.
